# 

**Published:** 1897-04-10

**Authors:** 


### EARLY EDITION.

**IMPORTANT NOTICE.**

**On account of the Easter Holidays, we beg to announce that we shall go to press with our issue of the 17th APRIL on TUESDAY NIGHT, the 13th INST. Advertisements intended for insertion in this issue, should therefore reach us BY NOON ON TUESDAY, the 13th inst.**

**Notices of Births, Deaths, and Marriages** are inserted in this column at a charge of 2s. 6d. for an announcement not exceeding four lines.

### NOTICE TO CORRESPONDENTS.

All MS., Letters, Books for Review, and other matters intended for the Editor should be addressed The Editor, "The Hospital," The Hospital Building, 28 & 29, Southampton Street, Strand, W.C.

The Editor cannot undertake to return rejected MS., even when accompanied by stamped directed envelope.

**Notice to Advertisers and Subscribers.**

All Advertisements, Orders for Copies of The Hospital, and Business Communications should be addressed to **THE MANAGER, (Not to The Editor),** The Hospital, The Hospital Building, 28 & 29, Southampton Street, Strand, London, W.C.

The Cost of Subscription, post free, is as follows:—Per week, 2½d.; per quarter, 2s. 8d.; per half-year, 5s. 3d.; per annum, 10s. 6d. Foreign:—Per annum, 15s. 2d.; payable, in all cases, in advance.

**NOTICE.—Vol. XXI. of "The Hospital" (October, 1896, to March, 1897), in handsome cloth binding, is on sale at the Office of this Journal, price 7s. 6d. Cases for binding the Numbers contained in this Volume, 1s. 6d. Index to Vol. XXI., 2½d., post free.**

**The Monthly Part for April is now ready. Price 6d., by post 8d.**

### BOOKS RECEIVED.

John Wright and Co., Bristol.

"The Swedish System of Physical Education." By Theodora Johnson.

Longmans, Green, and Co.

"Annual Charities Register and Digest." With an Introduction by C. S. Loch.

"Diphtheria and Antitoxin." By Nestor Tirard, M.D.

Adam and Charles Black.

"Who's Who," 1897. Edited by Douglas Sladen.

W. H. and L. Collingridge.

"The City of London Directory for 1897."

J. and A. Churchill.

"A Contribution to the History of the Respiration of Man." By William Marcet, M.D., F.R.S.

Page and Pratt.

"An Introduction to the Study of the Anthropoid Apes." By Arthur Keith, M.D.

**Periodicals and Pamphlets.**—Practitioner, Public Health, Bristol Medico-Chirurg. Journal, Girls' Own Paper, Boys' Own Paper, Medical Press, Sunday Hours, Leisure Hour, Sunday at Home, Woman's Signal, Electrical Review, Industries and Iron, The Gnyoscope, St. Ann's, London, Medical Record, The Sun, Literary Digest.

### The Student of Medicine.

### APPOINTMENTS.

E. G. E. Arnold, M.B., B.S., appointed Clinical Assistant to the Chelsea Hospital for Women. J. Aitken, M.B., C.M.Edin., appointed Medical Officer of the Workhouse of the Lancaster Union. T. R. Bailey, M.D.Edin, appointed Medical Officer for the Fourth District of the Wolverhampton Union. B. Bramwell, M.D., F.R.C.P.Edin., appointed Physician to the Edinburgh Royal Infirmary. F. R. Carter, M.R.C.S.Eng., L.S.A., appointed Medical Officer for the Sixth District of the Leeds Union. C. Corben, M.R.C.S., L.R.C.P.Lond., appointed Medical Officer for the Caldecott District of the Chepstow Union. R. Cox, L.R.C.P.Lond., M.R.C.S.Eng., appointed Medical Officer of Health to the Teme Rural Council. Dr. J. Crooks, appointed Medical Officer for the Seventh District of the Sudbury Union. A. F. Dixon, B.A., M.B., appointed Professor of Anatomy at the University College of South Wales and Monmouthshire. A. J. Grant, M.R.C.S, L.R.C.P., appointed Clinical Assistant to the Chelsea Hospital for Women. J. W. C. Herbert, L.R.C.P.Edin., M.R.C.S.Eng., appointed Medical Officer for the Pendlebury District of the Salford Union. A. O. Honnywill, L.R.C.S., L.R.C.P., L.M.Edin., appointed Surgeon to the Sutton District of the London, Brighton, and South Coast Railway Provident Society; also Medical Referee to the Prudential Assurance Company. A. W. Hughes, M.B., C.M.Edin., F.R.C.S.Eng., appointed Professor of Anatomy at King's College, London. Dr. Jeffrey, appointed Medical Officer for the Altofts District of the Wakefield Union. C. H. Kruger, M.B., C.M.Edin., appointed Clinical Assistant to the Chelsea Hospital for Women. W. W. Linington, F.R.C.S.Eng., appointed Resident Casualty Officer at the General Infirmary, Leeds. J. R. Lownds, B.A.Camb., L.R.C.P., L.R.C.S.Edin., L.F.P.S.Glasg., appointed Medical Officer of Health to the Brixworth Rural District Council. A. E. Lyster, M.R.C S.Eng., L.S.A.Lond., appointed Medical Officer and Public Vaccinator for the Fourth District of the Chelmsford Union. E. H. T. Nash, M.R.C.S., L.R.C P., appointed House Surgeon to the Ipswich and East Suffolk Hospital. G. J. Padbury, M.B, L.R C.P.Lond., M.R.C.S.Eng., appointed Medical Officer for the Hawkchurch District of the Axminster Union. S. W. Plummer, M.D.Durh., appointed Certifying Surgeon under the Factory Act for the Durham District. A. T. Rimell, M.R.C.S.Eng., L.R.C.P.Edin., appointed Medical Officer and Public Vaccinator for the Tydd, Sutton St. James, and Long Sutton Districts of the Holbeach Union. J. B. Ronaldson, M.D.St. And., F.R.C.S Edin., appointed Medical Officer of Health to the Dunbar Town Council. W. Roxburgh, M.B., C.M.Glasg., appointed Parochial Medical Officer for the Troon District. J. W. Smith, M.B.Edin., F.R C.S.Eng., appointed Honorary Anæsthetist to the Victoria Dental Hospital, Manchester. H. Tilley, M.D., B S.Lond., appointed Surgeon to the London Throat Hospital, Great Portland Street. F. C. Tothill, M.B., C.M.Edin., appointed Medical Officer for the Ashford District of the Staines Union F. H. Westmacott, F.R.C.S.Eng, L.R.C.P.Lond., appointed Honorary Anæsthetist to the Victoria Dental Hospital, Manchester. M. Young, M.B., C.M.Edin, D.P.H.Vict., appointed Medical Officer of Health for the Borough of Crewe.

